# Ten Simple Rules for landing on the right job after your PhD or postdoc

**DOI:** 10.1371/journal.pcbi.1007723

**Published:** 2020-04-02

**Authors:** Kuan-lin Huang

**Affiliations:** Department of Genetics and Genomic Sciences, Center for Transformative Disease Modeling, Tisch Cancer Institute, Icahn Institute for Data Science and Genomic Technology, Icahn School of Medicine at Mount Sinai, New York, New York, United States of America; Dassault Systemes BIOVIA, UNITED STATES

## Introduction

Freshly minted PhD and postdocs can often benefit from thorough guidance on career development and choosing the right job afterward. Several articles in the Ten Simple Rules series help you navigate this challenge, especially on selecting a postdoctoral position [1], considering a career in academia versus government [2], choosing between industry and academia [3], starting a company [4], and approaching a new job [5]. While these articles mostly include invaluable advice from senior leaders reflecting from decades of experience, I (having just gone through the process) wish to offer a complementary set of fresh-baked lessons.

During my PhD and a brief postdoc, I started a company, consulted part time, and participated in science-policy and teaching group activities. I then interviewed at the academia, biotech, and pharmaceutical companies before deciding on my current tenure-track position at the Icahn School of Medicine at Mount Sinai at the end of 2018. I am fortunate to have obtained a wide range of experience and cultivated networks of people to learn from (albeit most are limited to the United States). Here, I distill the ten rules into three sections representing distinct phases of landing on the right job—exploration, decision, and fulfillment—with practical tips (companion video: https://youtu.be/O6HZJgqhxA4).

## Exploration

*“Exploration is what you do when you don’t know what you’re doing*. *That’s what scientists do every day*.*”–Neil deGrasse Tyson*

### Rule 1: Know your values, goals, and priorities

Start by knowing yourself better. Life is a continuous journey of exploring and attaining one’s purpose. What impact do you want to have on the world? Try Steven Covey’s funeral exercise [6]: Picture attending your own funeral; what would you want your family, friends, colleagues, and others to say about you and what you have done? From the other angle, adopt Jim Collin’s hedgehog concept [7] for a company for yourself: Find how you can best contribute from the intersect between (1) what you are deeply passionate about, (2) what you can be the best in the world at, and (3) what you can get paid for.

You can also achieve tangible answers by asking yourself specific questions. Are you more driven by autonomy or order? Fortune or fame? Knowledge or utility? How much do you value spending personal time outside of work for yourself and the family, creating new knowledge and exercising intellectual autonomy, or translating science into products that directly affect consumers’ life? The other question that many people find helpful is as follows: If you were to pick one or a hybrid of multiple people as a model for your career, who would that be? What did that person do at the early stage of their career to help them become who they are?

At a practical level, reflect on what activity attracts or deters you from your work so far. Scientific research is often a job with multiple dimensions. You can dissect which aspects you particularly enjoy and thrive at. For example, do you enjoy the days when you are coding away or conducting experiments, or do you enjoy the days when you are writing manuscripts or drafting grants? Do you enjoy reading papers and planning experiments, or do you enjoy seeing the translational impacts that your research may have on others’ lives? Do you enjoy constant personal interactions, or do you prefer moments of solitude? You may not have a dichotomized answer for each of these exploratory questions. But pay attention to your preference. You can make decisions to optimize for it.

### Rule 2: Talk to everyone and see the possibilities

Your view of the possibilities constrains your choice. Expanding this view can help you identify the global maximum of reaching your potential. Being in the academic environment, many of you default to your PhD and postdoc advisors or other professors for career advice. While faculty may provide helpful thoughts for an academic career, we often possess limited insights on diverse “real-world” opportunities, considering that many of us have been at schools for our whole lives. Make sure you consult with the career center at your institution to explore your options; many now provide counselors with PhDs. Take every opportunity you have in networking events to talk with the majority of the PhDs who choose a nonacademic career path and learn about it. Listen, keep an open mind, and stay in touch. People are generally happy to share about their careers and willing to help those who are interested in following similar paths. And down the road, a referral—even from a weak tie—can get your first foot in the door in job applications.

### Rule 3: Gather the data

As scientists, you show the data to validate hypotheses and inform decisions. It should come as second nature to apply this evidence-based approach to your career too. Read books and journal articles written by diverse professionals to learn about different fields and the emerging trends you may be excited about. Additional information is also increasingly available to help evaluate your options. For example, job sites like Glassdoor post employee reviews, salaries, and interview tips. These sources can help you gather useful information that is not common knowledge. For example, a critical managerial change may be documented in recent employee reviews. Cross-validating such reviews with what I hear from my personal network or during on-site interviews, I find that many of them provide reasonable honest opinions on hard-to-gauge, yet important, aspects like organizational cultures.

### Rule 4: Conduct “mini-experiments” to try it out

One of the best ways to find out what you like is to do it. Many research institutions now have special-interest graduate and postdoc groups that enable you to try-out different careers. These groups can help you explore teaching, consulting, entrepreneurship, science policy, and other opportunities with varying levels of commitments. Not sure whether a consulting job suits you? Sign up for a quarterly consulting project. Have an itch for starting a company? Join a nearby incubator and explore start-up projects with like-minded individuals. You can also explore opportunities intersecting science and other fields through classes, seminars, and events at other schools such as the business school or the law school. Aside from the hands-on experience, these opportunities can help you expand your network and find advice from people of diverse professions.

### Rule 5: Sympathize with your future self

Sympathy is an essential attribute of a socialized human being. When it comes to making career choices, using thought experiments to sympathize with your future self may reveal insights. For example, imagine you are a professional a few years or even decades further along on the path you chose. Would you be proud of what you have achieved? Are those choices aligned with your values? As another practical example, think about the day-to-day routine of each job that you have now learned from prior explorations. Imagine yourself going through a typical day. Do you enjoy how your time is allocated to different categories of activities, especially those that would preoccupy most of your days? For example, would you enjoy grant writing as a junior faculty? Market research as a consultant? Coding as a computational biologist? Stretch and compress your current allocations for similar tasks to the extent demanded by the future job, and you may be able to see and feel more clearly whether you will strive in it.

## Decision

*“Do nothing*, *and nothing happens*. *Life is about decisions*. *You either make them or they're made for you*, *but you can't avoid them*.*”–Mhairi McFarlane*

### Rule 6: Evaluate the fit

Once you have gained a deeper understanding of yourself and the job, it is time to find whether the two align. Carefully examine the fit between your career goals, personality, and skillset and the organization’s mission, culture, and job requirements. Do your values align with the organization’s aims and goals? Carefully examine the organization’s mission statement and, sometimes more telling, its core economy-driving activity. For example, many internet technology companies derive values by optimizing advertisement views and clicks, which drives the economy (with the potential trade-off on consumer privacy and mental health). As another example, within the same industry, different pharmaceutical companies target different diseases, serve different populations, and deploy different pricing schemes. Your job is designed to advance the organization’s mission, which you will need to align to for long-term job fulfillment.

In evaluating personality fit, the unique challenges of the job often determine the organizational culture to a certain extent. For example, working in a large company likely requires you to execute excellence on your assigned task repeatedly. A job at a start-up probably involves a more dynamic role in which you will need to figure out the next challenges and solutions autonomously. A research-focused faculty job typically requires drafting of grant proposals relentlessly, especially in the early phase. Your role and the organization will evolve over time. Continue to evaluate, adjust, or—as needed—find a different fit.

### Rule 7. Get along with the five people around you

While each person is unique, it is natural to become more like the five people around you. The importance of getting to know your potential future teammates cannot be over emphasized. Most on-site interviews will arrange for you to meet with your prospective supervisors and colleagues. Make sure you interview them while they interview you. Are they happy? Can you imagine getting along with them? Do you share the same aspirations and levels of ambition? Would you enjoy a good time with them during work lunch and happy hours? If you could not gather enough information during the interview, follow up and politely ask whether you can ask more questions in a call or email.

### Rule 8: Plan out the dates for your transitions

Up until your PhD defense, all the start and end dates of school programs are set for you. You need to make the conscious switch that most future transition timings are jointly determined by you and the organization. A faculty job search can typically take a long duration with multiple on-site interviews. Industry and postdoc jobs tend to have faster turnarounds, and some may have strict deadlines for you to decide by. If your doctoral advisor holds conflicts in keeping you in the lab for longer, you may seek support from third-party faculty to determine if you have met the criteria of attaining a doctoral degree. If you are an international scholar, communicate early on with the international scholar office in your institution to make sure that your transition timing is aligned perfectly with your visas to avoid immigration issues. You need to take responsibility to align the dates for your unique circumstances.

### Rule 9: Don’t forget: Location, location, location

Now you have considered the organization, team, and timing, one often under-emphasized factor is the location. A job comes with the location at which you will likely live for at least several years of your life. It is where you will build your network and thus have a higher likelihood of staying long term. Would you prefer the cost-effective and spacious living at suburban locations or the dynamic lifestyles and job-hopping opportunities in big cities? How about places with warm sunshine year-round versus those with defined seasons? If you are moving with your family, what do they prefer? Each location is unique. I highly recommend that you spend extra time during on-site interviews to gain a brief living experience.

## Fulfillment

*“‘How does one become a butterfly*?’ *she asked pensively*.

*‘You must want to fly so much that you are willing to give up being a caterpillar*.*’”–Trina Paulus*

### Rule 10: Settle in, contribute, and keep learning

Transition is hard but also exciting. In the beginning, it is perfectly normal to be missing your previous lab and colleagues. Acknowledge that this is a natural process that most people go through and appreciate aspects of your new role and environment. There may also be times where you uncontrollably fantasize about the opportunities that you did not take. Identifying role models may provide some guiding lights—you can find plenty of accomplished individuals who stay with their grit and excel in one profession and just as many that switch tracks and contribute in multiple fields.

In the long term, finding meaning in your work is critical to a fulfilled life. Through knowing your values, goals, and priorities (Rule 1) and carefully evaluating the fit (Rule 6), you should be happily contributing to your and the organization’s shared mission with the right people in the right place (thanks to Rule 5, 7, 9). While the progress may have ups and downs, remember it took all your PhD and postdoc years to make a dent in expanding human knowledge. Given the right alignment, what you do every day shall accumulate to a substantial impact. Remember that landing on the job is part of a journey, not an end. You can always apply these rules to assess your situation and change courses.

Finally, as a scientist, you know that there is always plenty more to learn. Endowed with the knowledge and determination of a PhD, you are in a great position to contribute. But remember to maintain your gift of curiosity. Seek opportunities to grow. Continue to solve new challenges that are important to you and society. After all, a fulfilling career is not unlike a PhD—you ask an important question, experiment to solve it, and pursue the next one.
